# Neural-network classification of cardiac disease from ^31^P cardiovascular magnetic resonance spectroscopy measures of creatine kinase energy metabolism

**DOI:** 10.1186/s12968-019-0560-5

**Published:** 2019-08-12

**Authors:** Meiyappan Solaiyappan, Robert G. Weiss, Paul A. Bottomley

**Affiliations:** 1 Division of MR Research, Department of Radiology, Johns Hopkins School of Medicine, Park Bldg. 310, 600 N Wolfe St, Baltimore, MD 21287 USA; 2 Division of Cardiology, Department of Medicine, Johns Hopkins University, School of Medicine, Baltimore, MD USA

**Keywords:** Translational studies, Cardiac metabolism, Biomarker, Neural network, Heart failure, Phosphorus spectroscopy

## Abstract

**Background:**

The heart’s energy demand per gram of tissue is the body’s highest and creatine kinase (CK) metabolism, its primary energy reserve, is compromised in common heart diseases. Here, neural-network analysis is used to test whether noninvasive phosphorus (^31^P) cardiovascular magnetic resonance spectroscopy (CMRS) measurements of cardiac adenosine triphosphate (ATP) energy, phosphocreatine (PCr), the first-order CK reaction rate k_f_, and the rate of ATP synthesis through CK (CK flux), can predict specific human heart disease and clinical severity.

**Methods:**

The data comprised the extant 178 complete sets of PCr and ATP concentrations, k_f_, and CK flux data from human CMRS studies performed on clinical 1.5 and 3 Tesla scanners. Healthy subjects and patients with nonischemic cardiomyopathy, dilated (DCM) or hypertrophic disease, New York Heart Association (NYHA) class I-IV heart failure (HF), or with anterior myocardial infarction are included. Three-layer neural-networks were created to classify disease and to differentiate DCM, hypertrophy and clinical NYHA class in HF patients using leave-one-out training. Network performance was assessed using ‘confusion matrices’ and ‘area-under-the-curve’ (AUC) analyses of ‘receiver operating curves’. Possible methodological bias and network imbalance were tested by segregating 1.5 and 3 Tesla data, and by data augmentation by random interpolation of nearest neighbors, respectively.

**Results:**

The network differentiated healthy, HF and non-HF cardiac disease with an overall accuracy of 84% and AUC > 90% for each category using the four CK metabolic parameters, alone. HF patients with DCM, hypertrophy, and different NYHA severity were differentiated with ~ 80% overall accuracy independent of CMRS methodology.

**Conclusions:**

While sample-size was limited in some sub-classes, a neural network classifier applied to noninvasive cardiac ^31^P CMRS data, could serve as a metabolic biomarker for common disease types and HF severity with clinically-relevant accuracy. Moreover, the network’s ability to individually classify disease and HF severity using CK metabolism alone, implies an intimate relationship between CK metabolism and disease, with subtle underlying phenotypic differences that enable their differentiation.

**Trial registration:**

ClinicalTrials.gov Identifier: NCT00181259.

**Electronic supplementary material:**

The online version of this article (10.1186/s12968-019-0560-5) contains supplementary material, which is available to authorized users.

## Background

Since the mid-1980s numerous noninvasive localized phosphorus (^31^P) cardiovascular magnetic resonance spectroscopy (CMRS) studies have been performed on patients with heart disease to measure key energy metabolites–phosphocreatine (PCr) and adenosine triphosphate (ATP) [1]. ATP is the source of the cellular energy that fuels all cellular processes including myocardial contraction. PCr is the heart’s primary energy reserve, producing ATP from the phosphorylation of adenosine diphosphate (ADP) via the creatine kinase (CK) reaction: PCr + ADP < =>Cr + ATP, forming creatine (Cr) as a byproduct. The majority of human cardiac ^31^P CMRS studies use the PCr/ATP ratio as an index of this cardiac energy reserve [1]. Significant reductions in myocardial PCr/ATP are reported for a wide range of cardiac diseases including acute ischemia [2], dilated cardiomyopathy (DCM) [3–6], hypertrophic cardiomyopathy (HCM) [7–11], heart failure (HF) [3–11], valve disease [7, 8], obesity [12], diabetes [13] and in patients with heart transplants [14]. Although there is a trend for greater reductions in worse illness [4, 5, 8–10], the scatter and overlap amongst PCr/ATP values has nevertheless precluded its use as a metabolic biomarker for any specific condition or for grading disease severity, at least on an individual basis [1].

In the 1990s, methodologies that combine internal or external concentration references with magnetic resonance imaging (MRI) volumetry [10, 15–18], have enabled the measurement of absolute concentrations of human cardiac creatine kinase (CK) metabolites, revealing deficits in patients with cardiomyopathy, HF and myocardial infarction (MI) [6, 10, 11, 17]. In addition, the development of fast ^31^P saturation-transfer techniques over the past decade have provided measurements of the pseudo first-order kinetic reaction rate, k_f_ (in s^-1^) for the CK reaction [19, 20]. Reduced myocardial k_f_ was observed in patients with left ventricular hypertrophy (LVH) and HF, but not in those without HF [11]. This suggested that reductions in CK k_f_ might be specific to HF. By combining k_f_ and PCr concentration ([PCr]) measurements in the same cardiac ^31^P CMRS exam, the forward CK flux of ATP supplied to the heart can be obtained [6]. This is given by the product, k_f_ [PCr].

In nonischemic cardiomyopathy (NICM), CK flux declines with progression to HF [1, 21]. Indeed, the reduction in CK flux independently predicted cardiac events (HF hospitalization, transplantation, left-ventricular (LV) assist device requirement, cardiac death) in HF patients that occurred several years post-CMRS [22]. More recently it was shown that CK flux correlates with cardiac mechanical work [23], providing a mechanistic link between declining CK flux, mechanical work and cardiac events. While these findings support the ‘energy starvation hypothesis’ for HF [24, 25] and suggest that such measurements might index disease severity, the scatter among individual CK metabolite concentrations, k_f_, and flux data, again preclude the discrimination of disease type or severity on an individual basis, using any one ^31^P CMRS metric alone.

Today, trained machine learning techniques employing multi-layer neural networks are emerging as powerful tools for identifying and classifying a vast array of multi-parametric input data in applications ranging from bioinformatics to sociology to driving cars. Once designed, a neural network can be retuned and retrained as additional data become available, without exhaustively searching for the best classifier. Here, we apply neural-network analysis to test whether a combination of cardiac [PCr], [ATP], k_f_ and the CK flux supplying ATP to the heart, as measured noninvasively with ^31^P CMRS, can distinguish several common myocardial diseases and their clinical severity. The study includes–to the best of our knowledge–the entire known data pool of subjects for which complete sets of [PCr], [ATP], k_f_ and CK flux measurements exist [6, 11, 21–23, 26, 27]. The work tests the hypothesis that cardiac CK metabolism is altered in patterns that may be specific to different forms of heart disease and/or to the clinical severity of disease, specifically in HF. If true, cardiac CK metabolic data acquired with ^31^P CMRS could be used with a neural network classifier as a metabolic biomarker to gauge disease type and severity. Moreover, the existence of such relationships would link certain common clinical cardiac conditions to compromised CK energy supply in individual patients.

## Methods

### Subjects

Complete sets of cardiac [PCr], [ATP], k_f_ and CK flux data were obtained from 178 noninvasive cardiac ^31^P CMRS saturation transfer studies performed between 1999 and 2011 acquired with the approval of the Johns Hopkins University Institutional Review Board [6, 11, 21–23, 26, 27]. All participants provided written informed consent. Forty-five of the studies were from subjects 19–65 years-old (mean 40 ± 12 years) who were considered healthy with no history of heart disease, diabetes, or hypertension (controls). There were 109 patients, 20–72 years-old (mean 47 ± 12 years), with NICM including some with LVH who had no evidence of critical coronary disease (luminal stenosis > 50% as assessed by cardiac catheterization, computed tomography angiography, or positive stress nuclear or echocardiography) but had New York Heart Association (NYHA) class I-IV HF based on their clinical symptoms. A diagnosis of DCM was recorded for 38 of the 109 HF patients, but NYHA class was uncertain in one. For analysis, the HCM group included varying etiologies (pressure overload hypertrophy–LVH, genetic), with 23 cases documented among the 109 HF patients. In addition to the HF patients, there were 24 patients with no HF: 15 of these were 31–83 years who had anterior MI 7 weeks to 16 years prior to CMRS; the other 9 had HCM and were 41–80 years. The patient’s clinical diagnoses and NYHA class for HF severity were used as ‘ground truth’ for teaching and evaluating the accuracy of neural network classifications. The demographics of the study group are summarized in Table 1.

**Table 1:** Numbers of subjects (n) studied by noninvasive 1.5 T and 3 T ^31^P CMRS between 1999 and 2011 and their age (mean ±SD), gender, and clinical assessment at the time of CMRS

	1.5 T (*n* = 124)	3 T (*n* = 54)	HCM	DCM	NYHA class I	NYHA class II	NYHA class III	NYHA class IV
Control	31	14						
Age (yr)	40±8	40±18						
M/F	20/11	7/7						
HF-NICM	69	40	23	38	17	56	32	4
Age (yr)	48±11	46±14	48±13	49±12	44±13	48±12	47±12	44±5
M/F	43/26	16/26	11/12	29/9	10/7	27/29	20/12	2/2
HCM-no HF	9		9					
Age (yr)	59±13		59±13					
M/F	5/4		4/5					
Anterior MI-no HF	15							
Age (yr)	57±16							
M/F	12/3							

*HF* Heart failure, *NICM* nonischemic cardiomyopathy; *M/F* Male/Female, *HCM* Hypertrophic cardiomyopathy, *MI* Myocardial infarction, *NYHA* New York Heart Association (clinical classification for HF)

### CMR/CMRS methods

**Fig. 1:**
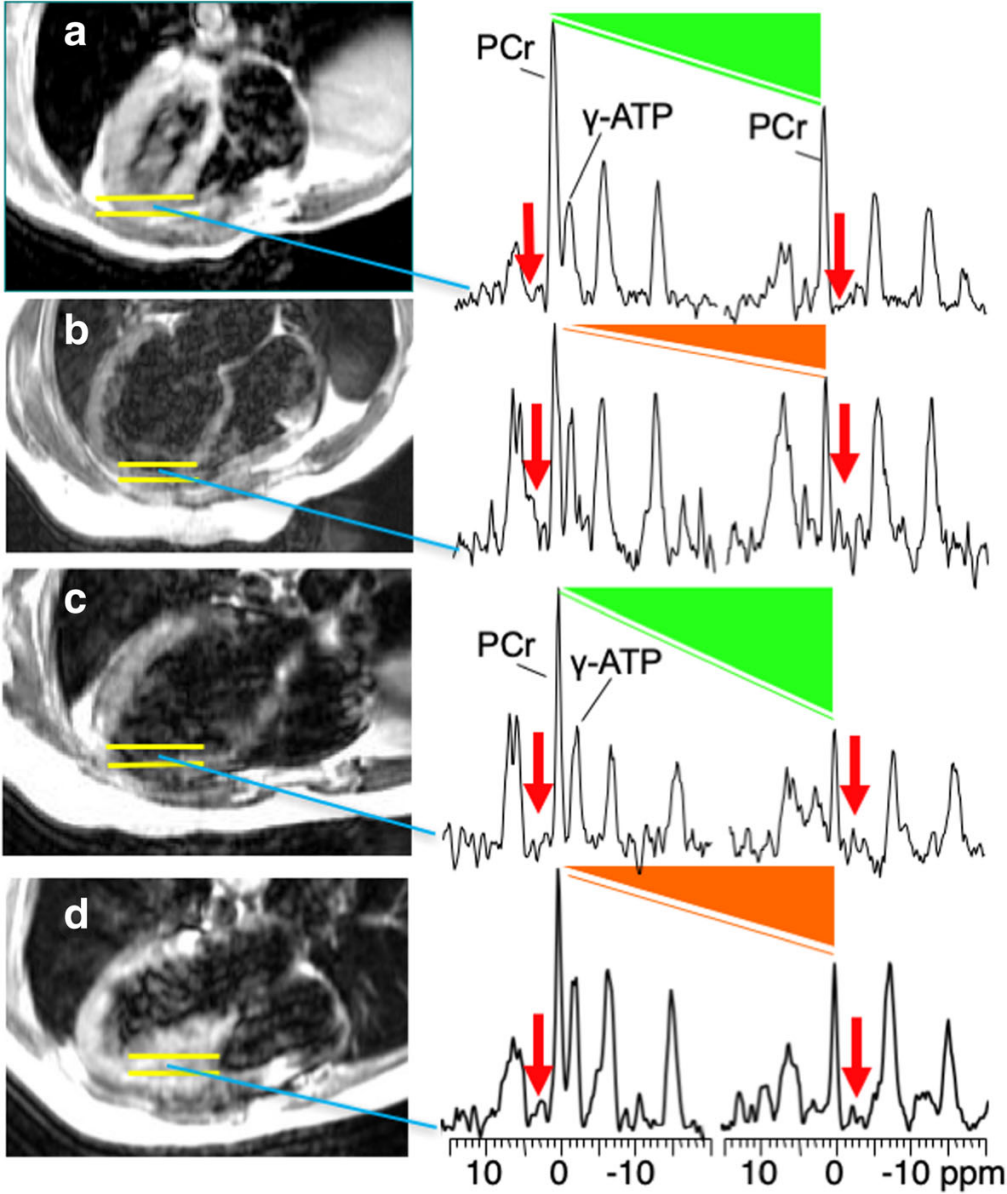
Examples of axial scout CMR (left) and two of the four FAST saturation transfer spectra (right) [19] from a single section in the anterior myocardium (yellow bars on CMR, left panel) of a healthy subject [6] (**a**); a patient with a dilated cardiomyopathy (DCM) and NYHA Class III heart failure (HF) [6] (**b**); one with anterior myocardial infarction (MI) and no HF [21] (**c**); and one with hypertrophic cardiomyopathy (HCM) and NYHA Class I HF[22] (**d**). Red arrows on the spectra denote the location of saturating irradiation which causes changes in the phosphocreatine (PCr) peak proportional to the creatine kinase (CK) pseudo first-order reaction rate, k_f_ (green triangles, no-HF; orange triangles, HF). In the absence of saturating radiation the PCr and adenosine triphosphate (ATP) signals are proportional to the concentrations, [PCr] and [ATP] which are measured with a reference concentration. For these subjects the ^31^P CK metabolic parameters of [PCr] in μmol/g, [ATP] in μmol/g, CK flux in µmol/g/sec and k_f_ in sec^-1^ were (12, 4.3, 3.3, 0.27), (8.5,6.9, 2.5, 0.29), (6.9, 4.2, 2.7, 0.39), and (6.1, 4.3, 2.8, 0.47) respectively

One hundred and twenty-four studies (31 healthy, 20–60 years; 94 patients, 22–83 years) were performed at 1.5 T using the ‘four-angle saturation-transfer’ (FAST) method [19] for measuring k_f_, and a combined internal (water) and external (phosphate) referencing method [15, 16] for measuring metabolite concentrations in the anterior myocardium and septum as identified by MRI [6]. Fifty-four subjects (14 healthy, 22–65 years, *p* = 0.99 vs 1.5 T; 40 HF patients, 20–69 years, *p* = 0.46 vs 1.5 T HF patients) were studied at 3 T using cardiovascular magnetic resonance (CMR)-guided ‘triple repetition time saturation-transfer’ (TRiST) [20] for measuring k_f_, and an external (phosphate) reference for the anterior myocardial metabolite concentrations [18]. One-dimensional chemical shift image localization was used with the same 1-cm spatial resolution for all acquisitions in each ^31^P CMRS protocol, with exam times ranging from 45 to 75 min. Figure 1 shows examples of raw CMR and ^31^P CMRS saturation transfer recordings at 1.5 T from healthy, DCM, anterior MI and hypertrophy subjects.

Cardiac [PCr], [ATP], and k_f_ were measured from CMRS peaks with signal-to-noise ratios (SNR) > 5 [22, 23] in the frequency domain, using simplex Gaussian fitting at 1.5 T and ‘Circle fit’ at 3 T. These analysis methods have been shown to produce indistinguishable results [28]. Each metabolic data set was comprised of an average of values from 2 to 4 sections of the anterior myocardial wall of each subject, corrected for partial saturation and spillover irradiation of k_f_ according to the latest published accounts [21–23]. No CMRS or clinical data were altered, filtered, reanalyzed, or corrected for this study, and no existing data sets were knowingly excluded. All data were treated as fully-corrected measurements of equal weighting and validity. The acquisition and data analysis protocols used for these studies were detailed in the original reports [6, 11, 21–23, 26, 27].

### Neural-network analysis

The machine learning and neural-network tools in MATLAB 2017A (Mathworks Inc., Natick, Massachusetts, USA) were used throughout. The MATLAB ‘Classification Learner App’ was first used to test whether non-neural network machine-learning approaches could be used to differentiate pooled HF data from pooled healthy and non-HF studies. The utility of the cubic ‘k nearest neighbor’ (KNN) cubic spline fitting; Gaussian ‘support vector machine’ (SVM); and ‘bagged decision tree’ (‘random forest’ bagging’, involving the random artificial duplication of data sets) methods were also tested, but their classification accuracy appeared limited to < 70% for each method with some evidence of over-fitting, so these methods were not pursued.

**Fig. 2:**
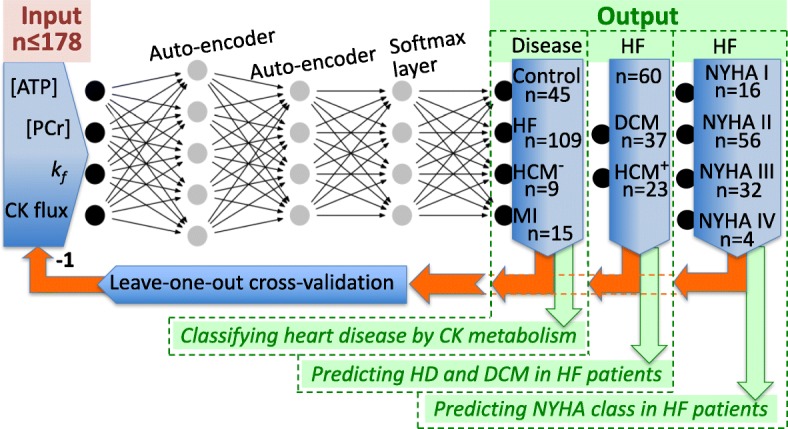
Schematic of the neural network used for classifying heart disease, heart failure (HF) type and New York Heart Association (NYHA) HF class, based on noninvasive measures of creatine kinase (CK) metabolism ([PCr], [ATP], k_f_ and CK flux), as implemented (HCM ± = hypertrophy with/without HF; MI = anterior myocardial infarction). Each grey dot represents a node with an input, a weight and a bias term. For the first layer, there are 25 parameters (4 weights plus 1 bias, times 5 nodes); the second layer has 24 (5 inputs times 4 nodes, plus 4 biases, 1 per node); the third ‘Softmax’ layer has 20 parameters (4 times 4, plus 4). The total number of parameters is thus 69 or about 10% of the 664 samples (166 times 4) used in the initial training for disease type (Table 2. This falls to 4% of the 4 × 388 = 1552 points when the synthetic data in Table 6 are included). The maximum number of training epochs was 8000

Seeking better performance, we designed a first neural network with two stacked MATLAB ‘auto-encoder’ layers, followed by a ‘softmax’ layer to classify the four-dimensional metabolic data (ATP, PCr, k_f_, and CK Flux) by disease type and HF severity. The auto-encoders reduce data dimensionality via compression (encoding) and decompression (decoding) functions that map the input data onto itself (hence ‘auto’) through back-propagation. The encoder’s output is the compressed representation of the input, which is either fed to the second auto-encoder layer, or to the network’s final output. Two auto-encoders were chosen to provide flexibility in dimensional reduction, while minimizing over-fitting that can result from either the rigidity of using a single auto-encoder, or the complexity introduced by more layers. Figure 2 shows a schematic of the model network as implemented.

The network was ‘trained’ by iteratively adjusting the weights and bias parameters associated with the individual network nodes using L2-regularization (performed internally within the MATLAB App ‘train’ function; L2 regularization parameter = 0.0025), and ‘leave-one-out’ cross-validation. The initial training utilized 166 of the data sets, omitting 12 patients who had both HCM and HF which would interfere with the fellow class of HCM without HF. Leave-one-out cross-validation can be considered equivalent to 166-fold cross-validation, as it is repeated 166 times with a different set of patient data omitted from each run to determine the corresponding accuracy and to adjust the hyper-parameters. The fluctuation in accuracy across the set of cross-validation outputs was used to fine-tune the hyper-parameters to achieve uniform (or ‘balanced’) accuracy of about 70% across all target classes. The network’s performance was then determined using all of the training data, with the results presented as a ‘confusion-matrix’, as ‘receiver operating curves’ (ROCs) which plot sensitivity versus (1-specificity) for the neural network classifications, and as ‘area-under-the-curve’ (AUC) analyses. The classification of data omitted from the training sets is reported separately.

Second, in order to determine which, if any, metabolic parameter(s) contributed most to disease classification and the potential impact of using fewer variables on prediction, we investigated the effect of the systematic elimination of input parameters from the network. After each omission, the network was redesigned to accommodate the reduced number of input parameters, while keeping the number of layers the same. The redesigned network was re-trained to optimize the hyper-parameters for the network using leave-one-out cross-validation, and the final performance analyzed to determine the change in accuracy and AUC.

Third, we tested whether a CK metabolism-based neural network could distinguish subgroups of the HF patient group who were also documented with DCM (*n* = 37, with the case of uncertain NYHA class excluded from the training set) and HCM (*n* = 23, now including the 12 HCM cases with HF omitted from the initial analysis). This (third) network was trained and optimized using the same methods described above.

Fourth, we tested whether CK metabolism could distinguish the degree of clinical HF severity as indexed by NYHA class among the subgroup of 109 HF patients. Here we used a (fourth) two-stage serial network because the closer proximity of the NYHA class II and III metabolic data (vs. the NYHA class I and class IV data) compromised network balance when all four classes were fitted simultaneously. Thus the HF data were first classified into three classes: NYHA class I; NYHA classes II plus III combined; and NYHA class IV. The output of the combined NYHA classes II and III of the first network stage, were input to a second neural network classifier to distinguish the two. Omitting the uncertain NYHA case, this study included 108 of the 109 HF cases.

### Confounding factors

Because the distribution of patients studied at 3 T differed from those at 1.5 T, we further tested whether the classification of heart disease from CK metabolic data was confounded by the use of the different methodologies rather than their underlying metabolic characteristics. For this, the performance of the first network that was trained to classify the control, HF and non-HF patients using the combined 1.5 and 3 T data, was tested separately on the 1.5 T and the 3 T data sets without retraining. Next, we retrained and re-optimized the network using the 1.5 T data alone, and re-tested its performance on the 1.5 T data and on the pooled 1.5 T plus 3 T data.

To test the effects of unbalanced sampling of both disease type and HF NYHA class on neural network performance without discarding any patient data, we used the ‘synthetic minority over-sampling technique’ (SMOTE) to augment the original class sets with data randomly interpolated between nearest neighbors in parametric space [29, 30]. Each synthetic point was randomly interpolated from one of five nearest-neighbor pairs of each of the three independent parameters, [PCr], [ATP] and k_f_. CK flux, which is a dependent parameter, was derived from the product of the new [PCr] and k_f_ data. Points were added until the disease class-size matched that of the majority training class (HF, *n* = 97). Points in excess of the targeted class size that arose from the multi-fold interpolation were omitted as outliers, as assessed by the original neural network, analogous to the ‘generative adversarial neural network technique’ [31]. Similarly, NYHA class size was balanced by SMOTE interpolation to match that of the majority Class II HF (*n* = 56), after discarding the Class IV data (*n* = 4) as too limited for this purpose. Given the larger data sets generated by this process, the neural networks for disease type and HF severity were retrained with the synthetically-balanced data sets using ‘5-10 fold cross-validation’ (that is, an 80–90% training versus 20–10% testing data split) instead of leave-one-out cross-validation. The performance was then re-assessed. Finally, the performance of the balanced retrained network was tested with the original data; and the network trained with the original data was tested with the augmented HF data.

## Results

Table 2 presents the confusion-matrix documenting the accuracy of the first network for classifying healthy and heart disease patients. The results show an overall accuracy of 84% for discriminating heart disease solely on the basis of CK metabolism alone. The network correctly distinguished 90% of the HF data, and about 80% of the other classes except for MI, which was difficult to differentiate from HF (accuracy ~ 70%). It predicted the primary diagnosis of HF in the 12 HCM cases that were omitted from the training set (and Table 2), with 100% accuracy.

**Table 2:**
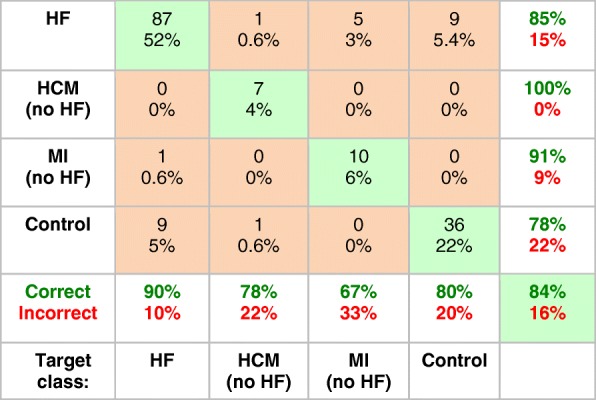
Confusion matrix for the prediction of heart disease for the (first) neural network using the four CK metabolic parameters, [PCr], [ATP], k_f_, and CK flux). The numbers at the top of the boxes are the number of cases classified correctly (green boxes), or misclassified (orange boxes): their sum is the total classified (166 = 178–12 HCM patients who had HF and were omitted from the training set). The percentages of the total are listed below the numbers (eg, for HF, 87/166 = 52% of the sample). At bottom, the % correct (true positive rate, green bold font) and incorrect (false negative rate, red bold font) are summarized for each output disease class (HF, HCM, MI and Control). For example, in the first column 87 HF cases were correctly classified. An additional 10 HF cases were incorrectly assigned as non-HF (1 as MI and 9 as Controls). Thus 87/97 = 90% are correct and 10% are incorrect. The percentages in the right-hand column (positive predictive value, green font; false discovery rate, red font) include subjects from all classes. For example, a total of 102 cases were classified by the network as HF. This comprises 87 true HF cases, plus 1 HCM, 5 MI and 9 Controls all misclassified as HF. Thus, 87/102 = 85% are correct. Summing the diagonal, 140/166 = 84% of cases are correctly classified overall

**Fig. 3:**
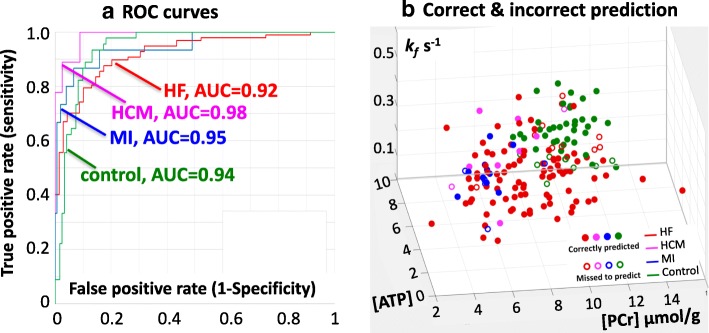
(**a**) Receiver operating curve (ROC; the true positive rate or sensitivity vs. the false positive rate, or 1-specificity) analyses of the network’s classification of healthy control subjects (green); patients with HF (red); and those with MI (blue) and HCM (magenta), both without HF. The areas under the curve (AUC) are 0.94, 0.92, 0.95 and 0.98, respectively. (**b**) Three-dimensional (3D) plot of the independent variables [PCr], [ATP] and k_f_ depicting the network’s correct (solid symbols) and incorrect classifications


Figure 3(a) illustrates the results of the ROC analyses of the (first) network’s classification of each disease using the four CK metabolic parameters. The AUCs are all greater than 90%. Figure 3(b) is a three-dimensional (3D) plot of the independent variables [PCr], [ATP] and k_f_ depicting the network’s true and false classifications. A 3D video plot (see Additional file 1) shows that the fitted volumes associated with each output disease type are intertwined on the [PCr]–[ATP] axis (diagonal), indicating that the PCr/ATP ratio, by itself, is a poor discriminator of disease type.

**Additional file 1**: CK metabolism disease classifier and fitting volumes in 3D. (MP4 3733 kb)


**Fig. 4:**
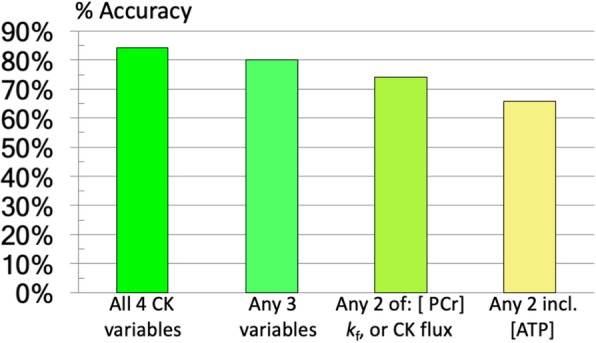
The overall accuracy of the network after successively eliminating each CK metabolic parameter and re-tuning/optimizing it. All variables appear to contribute to successful discrimination

**Fig. 5:**
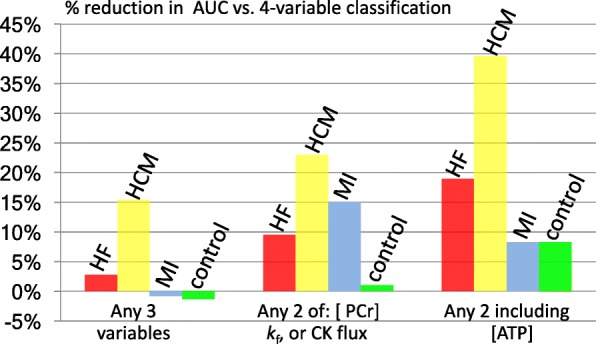
The effect of omitting variables on the ROC analysis for each disease class: control, HF; and MI and HCM without HF. The vertical axis is the net % reduction in area-under-the ROC-curve (AUC) as compared to the (first) 4-variable network that uses all of the [PCr], [ATP], k_f_ and CK flux data. The horizontal axis denotes which of the four are included in the ROC analysis

The effect of successively eliminating in turn, each CK metabolic parameter and re-tuning the network in the second set of studies is shown in Fig. 4. All variables contributed to discrimination, but [ATP] contributed least to overall accuracy. The effect of omitting metabolic variables on the ROC–AUC analysis, for each disease type (Fig. 5) shows that: (i) successively removing variables reduces the AUC; (ii) patients with HCM but no HF were hardest to classify; and (iii) [ATP] provided the least added-value as a discriminator, as evidenced by the fact that including [ATP] instead of any other variable produced the greatest loss in AUC.

Table 3 shows the confusion matrix for the (third) network designed to distinguish HF patients documented with DCM from those with HCM, based just on their CK metabolism. Again an accuracy of about 80% was achieved. The network predicted the one DCM subject whose NYHA class was uncertain as class II HF (presently not confirmable), with a 50/50 chance of being either DCM or HCM.

**Table 3:**
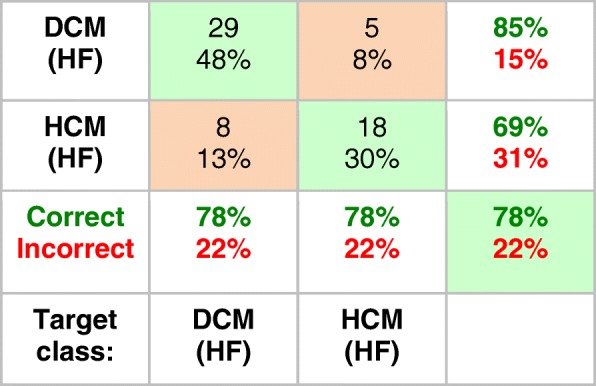
Confusion matrix for predicting HCM (*n* = 23 cases) and DCM (*n* = 37, excluding one with uncertain NYHA class) among HF patients with NYHA class ≥ I using a neural network based on the four CK metabolic parameters, [PCr], [ATP], k_f_, and CK flux. The numbers at the top of the boxes are the number of cases classified correctly (green boxes) or misclassified (orange boxes), with the percentages of the total listed below. The % correct (green, bold font) and incorrect (red, bold font) are summarized for each output class (see Table 1 caption for interpretation)

Table 4 presents the confusion matrix results for classifying NYHA HF severity using the (fourth) CK metabolism-based neural network classifier. The overall accuracy for discriminating NYHA HF class on the basis of CK metabolism alone was 86%, with the caveat that the number of NYHA class IV patients is very limited.

**Table 4:**
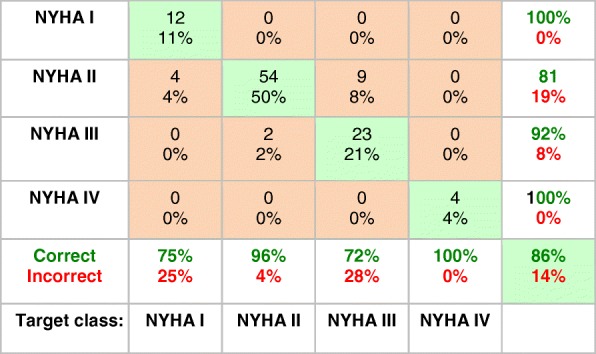
Confusion matrix for the prediction of NYHA class I-IV for HF among HF subjects using a two-stage neural network and the four CK metabolic parameters [PCr], [ATP], k_f_, and CK flux as input. The numbers at the top of the boxes are the number of cases classified correctly (green boxes), or misclassified (orange boxes), totaling 108 cases excluding the one case with uncertain NYHA class (See text). The percentages of the total are listed below the numbers. The % correct (green, bold font) and incorrect (red, bold font) are summarized for each output NYHA class (see Table 1 caption to guide interpretation)

When all the 3 T data are omitted from the first neural network that was used for Table 2 in order to assess whether the results are confounded by differences in the 1.5 T and 3 T methodologies, the accuracy of the resulting confusion matrix (Table 5a) improved slightly to 86% (vs 84% in Table 2). Omitting the 1.5 T data instead of the 3 T data yielded a similar 82% accuracy using this same un-retuned (first) network (Table 5b). Retraining and optimizing this network for just the 1.5 T data alone, improved the overall accuracy to 90% (Table 5c). This may be due to some systematic differences (including variability in scatter) between the 1.5 T and 3 T data and/or differences in the sub-groups sampled.

**Table 5:**
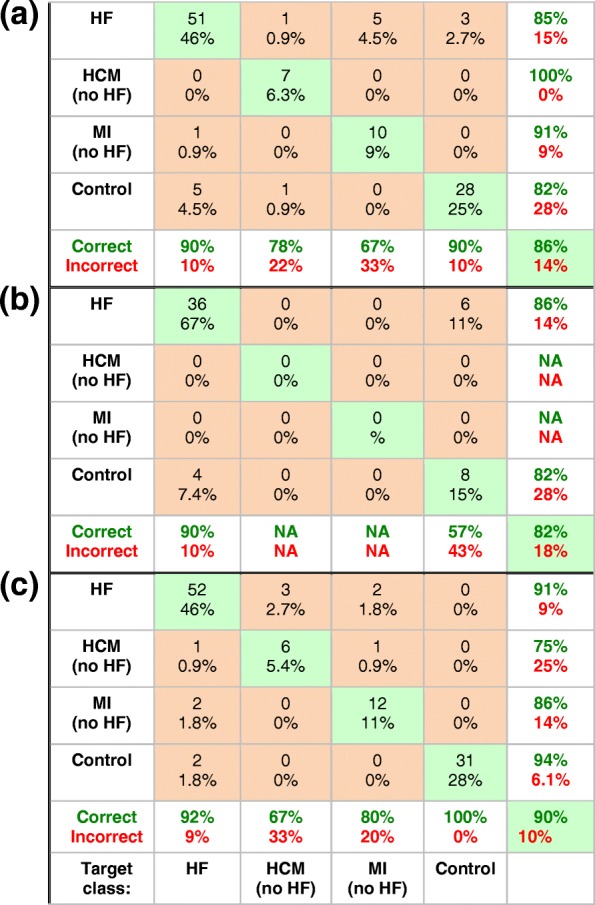
Confusion matrices for the prediction of heart disease for the neural network used in Table 2, applied to: (a) the 1.5 T data alone (*n* = 112 omitting 12 interfering cases with both HD and HF); and (b) the 3 T data alone (*n* = 54 cases). (c) Confusion matrix after retuning and optimization using only the 1.5 T data. (NA = not applicable due to absence of 3 T data for that class)

Rebalancing the network used for classifying disease with randomly interpolated data to equalize class size to that of HF, resulted in the same overall accuracy of 84% (Table 6a). This reflects an improvement by about 10% in the accuracy of detecting the formerly lesser-sampled LVH, MI and control groups, at the expense of HF patients whose accuracy declined by 14%. Similarly, rebalancing the network for distinguishing HF severity by augmenting the NYHA Classes I and III data to match the Class II data pool-size, resulted in essentially the same overall accuracy (85% vs. 86% in Table 4), with performance improving for Classes I and III, at the expense of Class II (Table 6b). Applying the balanced retrained network to the original data reduced overall performance a little to 78%, mainly to the detriment of the small HCM non-HF group (accuracy, 67%). Finally, when the network that was trained with the original data from Table 2, was plied with the entire set of augmented HF data (*n* = 168), its overall accuracy for detecting HF was 85%.

**Table 6:**
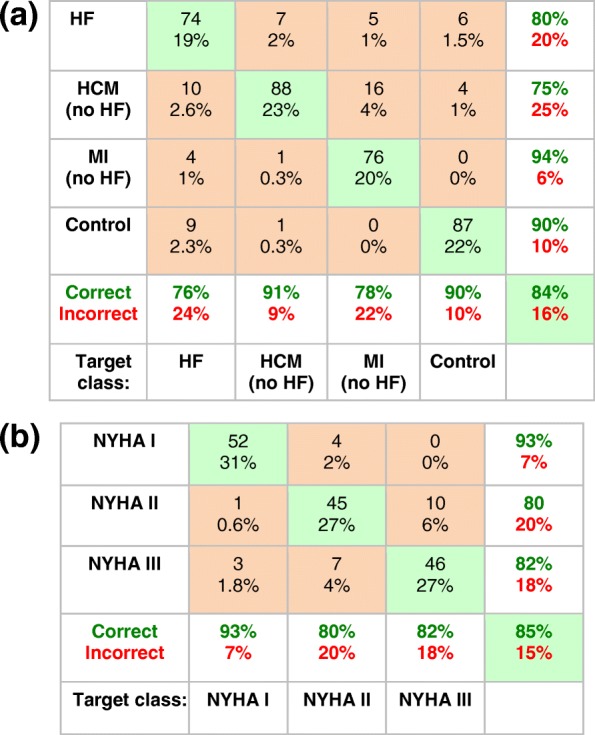
Confusion matrices for predicting heart disease and HF severity based on neural networks retrained with the augmented synthetic [PCr], [ATP] and k_f_ data to balance class size. (a) Results from the network as in Table 2, retrained with 388 cases comprised of groups of 97 with HF, and 97 synthetically-augmented cases of HCM, MI and control subjects. (b) Results from the network as in Table 4, retrained with 168 cases of HF comprised of 56 patients with NYHA Class II severity and two synthetically-augmented groups of 56 each, with NYHA Class I and III

## Discussion

This is the first study to apply neural network techniques to test whether cardiac CK energy metabolism can differentiate the type of myocardial disease and the severity of disease in individual patients. The source data comprised all of the known complete sets of individual cardiac [PCr], [ATP], k_f_ and CK flux data from healthy subjects and heart disease patients extant in the world that we know of [1, 6, 11, 21–23, 26, 27]. The results show that a three-layer neural-network can differentiate healthy, HF, non-HF MI and HCM cardiac diseases with an accuracy of 84% and an AUC of > 90% for each category, based on their CK metabolism alone (Table 2, Fig. 3). Among HF patients a neural network was able to differentiate DCM and HCM (Table 3), as well as the severity of HF as indexed by NYHA class (Table 4), both with about 80% or higher overall accuracy. This suggests that CK metabolic data acquired noninvasively with resting-state cardiac ^31^P CMRS, could be used with a neural network classifier as a metabolic biomarker to stratify disease type and severity with clinically-relevant accuracy. Moreover, from a mechanistic standpoint, the fact that a neural network can classify disease based on CK metabolism alone, means that there must exist heretofore unidentified differences in CK metabolism that are specific to disease type and severity which enable the network to do so.

Indeed there was no one unique disease-specific CK-metabolic defect, as evidenced by the incremental effect of successively removing each parameter and retuning the network (Fig. 4). Instead, nuanced variations in multiple parameters permitted accurate neural network discrimination. The elimination analysis showed that [PCr] and k_f_ were the first and second most important input parameters for classifying heart disease in the neural network (Figs. 4 and 5). The pair, [PCr] and k_f_, was better than CK flux alone, but using all three, namely CK flux, k_f_, and [PCr], was better still. This is unexpected because CK flux is not independent, being the product of k_f_ and [PCr] as noted earlier. In order for CK flux to add such value as a classifier compared to its component factors, k_f_ and [PCr] must vary in a co-dependent way that is exacerbated by disease. This is consistent with the observation that CK flux independently predicted cardiac events whereas individual k_f_, [PCr] (and PCr/ATP) measures alone, did not [22]. [ATP] was the least important parameter for discriminating disease, an observation likely reflecting the tight control of [ATP] that is required to preserve cellular viability. With [ATP] as the ‘last to go’, the CK reaction, oxidative phosphorylation, glycolysis and other metabolic pathways apparently make whatever adjustments that are needed to preserve [ATP] and prevent demise.

## Limitations

The main limitation of this work is the number of studies, particularly the NYHA class IV HF, and non-HF HCM and MI sub-categories. Although all extant complete data sets were included, they derive from research studies that were not designed for the specific investigation reported herein. In that respect the study might be considered as blinded compared to the prospective acquisition of data to test a preconceived hypothesis. Unfortunately, the availability of new data to test the potential of this cardiac ^31^P CMRS quartet to serve as a metabolic biomarker for clinical disease, is presently limited to the extent that cardiac ^31^P MRS is not yet a clinical procedure.

The synthetic augmentation of metabolic data by 2.3-fold for disease type and by 1.7-fold for HF severity using the SMOTE method, provided results that were consistent with the smaller original data sets. This suggests that the overall level of performance realized here should be reasonably representative of what is achievable with the addition of ^31^P CMRS data of comparable quality from future studies. Nevertheless, the primary purpose of data augmentation was to balance class-size to avoid over-fitting and/or bias of the neural network towards a majority class. Indeed, its overall effect was to smooth out performance differences between the majority and lesser-sampled classes (Table 6 vs. Tables 2 and 4), which was the intended purpose [29]. There is no substitute for original data, especially when the underlying functional inter-relationship between metabolic parameters, disease type and severity is not known. That the successive dropping of metabolic parameters degraded predictive accuracy (Fig. 4), means that [PCr], [ATP], k_f_ and CK flux have to be treated as a parametric quartet. Thus in general, network performance might not be expected to survive simulations that involve independent randomization of the component parameters, at least until the nature of their mechanistic link is better understood. Disease heterogeneity is a potentially confounding factor as hypertrophy, DCM and MI are common co-morbidities that underlie HF. They would therefore be expected to confound the neural network’s ability to distinguish them. In that sense, the accuracy with which the network was able to discriminate disease type as demonstrated here, is quite surprising. Also surprising was its ability to differentiate NYHA class, which involves a degree of subjectivity in clinical assessment. Indeed all of the network’s misclassification errors involved sliding into an immediately adjacent NYHA class (Table 4).

Other potential confounding variables were age and gender differences, and the use of different 1.5 and 3 T methodologies among sub-groups of the study population. As can be seen in Table 1, the former are fairly minor with the ages of control and HF patients being around 40–50 years. Although the MI and non-HF HCM patients were older, these patients had normal k_f_ and [ATP] when compared with their original study controls [11, 21]. Nevertheless, a trend for reduced metabolite concentrations (and probably PCr/ATP) with age over 40 [1] has been reported elsewhere and should be noted. Regarding methodological differences between the 1.5 and 3 T CMRS protocols, the re-analysis of separated 1.5 T and 3 T data should eliminate any such confounding effects (Table 5). Indeed, the ability of the neural network to classify disease based on CK metabolism alone was unimpaired for those diseases for which CK metabolic data were available from both 1.5 T and 3 T methodologies. These results can only further support the primary conclusion that a neural network can differentiate individual disease type and severity based on CK metabolism alone.

## Conclusion and clinical implications

Neural network analysis employing only cardiac CK metabolic parameters can accurately classify cardiac disease and severity, suggesting a role for noninvasive quantitative ^31^P CMRS as a metabolic biomarker. In HF, the degree of compromise in CK energy supply as identified by the neural network, predicted the clinical severity of HF symptoms in individual patients. The ability to accurately classify disease based on CK metabolism suggests that altered CK metabolism is an intrinsic and nuanced factor in many common heart diseases. If the relationship between compromised CK metabolism and clinical severity is causal, then therapies that increase CK energy supply could well reduce symptoms and support a role for the CK reaction as a potentially important new therapy target [22, 26].

## Data Availability

Data generated during this study are included in this published article, the supplementary file, ^*ClinicalTrials.gov*^ (Identifier, NCT00181259), and the cited references.

## Abbreviations

3D: Three dimensional
ADP: Adenosine diphosphate
ATP: Adenosine triphosphate
AUC: Area-under-thecurve
CK: Creatine kinase
CMR: Cardiovascular magnetic resonance
CMRS: Cardiovascular magnetic resonance spectroscopy
Cr: Unphosphorylated creatine
DCM: Dilated cardiomyopathy
FAST: Four-angle saturation transfer
HCM: Hypertrophic cardiomyopathy
HF: Heart failure
KNN: K nearest neighbor
LV: Left ventricle/left ventricular
LVH: Left ventricular hypertrophy
MI: Myocardial infarction
MRI: Magnetic resonance imaging
NICM: Non-ischemic cardiomyopathy
NYHA: New York Heart Association
PCr: Phosphocreatine
ROC: Receiver operating curve
SD: Standard deviation
SMOTE: Synthetic minority over-sampling technique
SNR: Signal- to -noise ratio
SVM: Support vector machine
T: Tesla (unit)
TRiST: Triple repetition time saturation-transfer
